# Dynein Light Chain 1 (DYNLT1) Interacts with Normal and Oncogenic Nucleoporins

**DOI:** 10.1371/journal.pone.0067032

**Published:** 2013-06-26

**Authors:** Nayan J. Sarma, Nabeel R. Yaseen

**Affiliations:** Department of Pathology and Immunology, Washington University School of Medicine, Saint Louis, Missouri, United States of America; University of Pittsburgh Cancer Institute, United States of America

## Abstract

The chimeric oncoprotein NUP98-HOXA9 results from the t(7;11)(p15;p15) chromosomal translocation and is associated with acute myeloid leukemia. It causes aberrant gene regulation and leukemic transformation through mechanisms that are not fully understood. NUP98-HOXA9 consists of an N-terminal portion of the nucleoporin NUP98 that contains many FG repeats fused to the DNA-binding homeodomain of HOXA9. We used a Cytotrap yeast two-hybrid assay to identify proteins that interact with NUP98-HOXA9. We identified Dynein Light Chain 1 (DYNLT1), an integral 14 KDa protein subunit of the large microtubule-based cytoplasmic dynein complex, as an interaction partner of NUP98-HOXA9. Binding was confirmed by *in vitro* pull down and co-immunoprecipitation assays and the FG repeat region of NUP98-HOXA9 was shown to be essential for the interaction. RNAi-mediated knockdown of DYNLT1 resulted in reduction of the ability of NUP98-HOXA9 to activate transcription and also inhibited the ability of NUP98-HOXA9 to induce proliferation of primary human hematopoietic CD34+ cells. DYNLT1 also showed a strong interaction with wild-type NUP98 and other nucleoporins containing FG repeats. Immunofluorescence analysis showed that DYNLT1 localizes primarily to the nuclear periphery, where it co-localizes with the nuclear pore complex, and to the cytoplasm. Deletion studies showed that the interactions of the nucleoporins with DYNLT1 are dependent predominantly on the C-terminal half of the DYNLT1. These data show for the first time that DYNLT1 interacts with nucleoporins and plays a role in the dysregulation of gene expression and induction of hematopoietic cell proliferation by the leukemogenic nucleoporin fusion, NUP98-HOXA9.

## Introduction

The nucleoporin NUP98, a component of the nuclear pore complex, is characterized by numerous FG (phenylalanine-glycine) repeats and plays important roles in nucleocytoplasmic transport [1], [2]. The N-terminal half of NUP98 contains these FG repeats that are crucial for its role in nucleocytoplasmic transport [1], [3], [4]. At least 28 chromosomal rearrangements affecting the *NUP98* gene, have been reported in many hematopoietic malignancies, particularly acute myeloid leukemia (AML) [5]. Notably, the N-terminus containing the FG repeats is retained in all the oncogenic fusion proteins resulting from *NUP98* gene rearrangements. Of the NUP98 fusion partners, 10 are homeodomain transcription factors, including HOXA9 [5]. The best-characterized NUP98-homeodomain fusion is NUP98-HOXA9 [6], [7], [8], [9], [10], [11], [12], [13], [14], [15], [16], [17], [18], [19], [20]. NUP98 fusions including NUP98-HOXA9 have been shown to induce aberrant proliferation and to disrupt differentiation in both human and mouse hematopoietic precursors [9], [14], [17], [19], [21], [22]. Proteins that interact with NUP98-HOXA9 and play a role in downstream gene regulation and leukemic transformation have not been well defined. We have previously shown that amino-terminal enhancer of split (AES) interacts with NUP98-HOXA9 and cooperates with it in transcriptional dysregulation and cell transformation [16]. NUP98-HOXA9 also interacts with CBP/p300, HDAC, and CRM1, resulting in transcriptional activation, transcriptional repression, and inhibition of nuclear export, respectively [7], [12], [13], [18], [19], [23], [24].

This study was aimed at identifying other NUP98-HOXA9-interacting proteins and determining their role in the dysregulation of gene expression by NUP98-HOXA9. Traditional yeast two-hybrid assays based on intranuclear transcriptional activation are likely to show a high number of false positives because of the transactivating properties of the FG repeat-rich N-terminus of NUP98-HOXA9 [13], [25]. On the other hand, the Cytotrap yeast two-hybrid method is based on interactions occurring in the cytoplasm and is not affected by the transactivating properties of the bait protein. Using this technique, we identified DYNLT1, an integral protein subunit of the large microtubule-based cytoplasmic dynein complex [26], [27], [28], as a novel interaction partner of NUP98-HOXA9 that is important for the ability of NUP98-HOXA9 to dysregulate gene transcription.

## Materials and Methods

### K562 cDNA Library and Plasmid Construction

A K562 cDNA library was constructed as described previously [16]. Briefly, total RNA was isolated from 32×10^6^ K562 cells (ATCC) using RNAaqueous Kit (Ambion) according to the manufacturer’s instructions. PolyA RNA was purified using PolyA Purist Kit (Ambion) from the total RNA (350 µg) according to the manufacturer’s instructions. A total of 5 µg polyA RNA was recovered and a cDNA library was synthesized and subcloned into pMYR XR vector using the Cytotrap XR library construction kit (Stratagene). Control plasmids for the two-hybrid assay were provided with the kit. The bait plasmid pSOS-HA-NUP98-HOXA9 was constructed by subcloning HA-tagged NUP98-HOXA9 in-frame from pcDNA-HA-NUP98-HOXA9 [12], [16]. pGEX6P1-HA-NUP98-HOXA9 and pGEX6P1-HA-HOXA9 were constructed by subcloning from pcDNA-HA-NUP98-HOXA9 and pcDNA-HA-HOXA9 [12]. pGEX6P1-DYNLT1 and pcDNA-DYNLT1 were constructed by subcloning PCR-amplified DYNLT1 cDNA from pMYR-DYNLT1. NUP98-HOXA9 deletion mutants, pGL4.11-KBTBD10, and full length and deletion mutants of NUP62 and NUP153 are described elsewhere [18], [19], [29]. pGEX6P1 expressing DYNLT1 N-terminus or C-terminus, and pcDNA expressing DYNLT1 N-terminus or C-terminus were subcloned from pGEX6P1-DYNLT1. All PCR products were verified by DNA sequencing.

### Cytotrap Yeast Two Hybrid Analysis

Cytotrap yeast two-hybrid analysis was carried out following the manufacturer’s instructions. Briefly, the pMYR vectors containing the K562 cDNA library and pSOS-HA-NUP98-HOXA9 were co-transformed into the temperature sensitive cdc25H yeast strain, plated into selective medium containing glucose as the carbon source and incubated at room temperature till colonies appeared. The colonies were replica-plated into positive selective medium containing galactose as the carbon source and incubated at 37°C till visible colonies appeared. Temperature resistant cdc25 yeast colonies growing at 37°C were isolated, patched into new plates containing selective media, grown, and replica plated again to confirm the positive growth and eliminate false positives. Cells from the patches were grown in liquid medium and pMYR vectors containing the positive clones were isolated. The isolated plasmids were transformed and amplified in *E. coli* and DNA was isolated. The purified DNA was sequenced using primers on both sides of the cDNA insert regions. The cDNA inserts were identified using the National Center for Biotechnology Information blast application (http://blast.ncbi.nlm.nih.gov/Blast.cgi).

### Recombinant Proteins

Glutathione S-transferase (GST), as well as GST-tagged HA-NUP98-HOXA9, HOXA9, and DYNLT1 were produced from the pGEX-6P1 vector in *E. coli* BL21 (DE3) bacteria and purified using glutathione-Sepharose 4B beads (GE Healthcare). Recombinant DYNLT1 was isolated by digestion with Pre-Scission protease (GE Healthcare).

### Protein Binding Assays

Full length NUP98, NUP98-HOXA9 and its deletion mutants, as well as full length NUP62 and NUP153 and their deletion mutants, were produced using the TnT T7 quick coupled transcription/translation system (Promega) in the presence of Tran35S-label (MP Biomedicals). Protein binding assays were carried out as described previously [29]. Briefly, for a binding reaction, 10 µl of beads of immobilized recombinant protein (GST control, or GST- DYNLT1, or GST- DYNLT1 N or C terminus) were incubated for 1 h at 4°C with 34 µl of a mixture consisting of *in vitro* translated protein, transport buffer-Tween 20 (TB-T, which consists of 20 mm HEPES-KOH, pH 7.4, 110 mm potassium acetate, and 2 mm MgCl2 with 0.1% Tween 20) and 0.5 mg/ml Pefabloc (Roche). The unbound fraction was removed and the beads were washed three times in cold TB-T. One fourth of the unbound and all of the bound fractions were visualized by SDS-PAGE and autoradiography.

For binding assays using all recombinant proteins, purified recombinant DYNLT1 was incubated with immobilized GST, GST-NUP98-HOXA9, or GST-HOXA9 proteins in 34 µl mixtures in TB-T and processed as described above. Bands were visualized by staining with Coomassie blue.

### DYNLT1 Antibody Preparation

Recombinant DYNLT1 protein was purified as described above and was submitted to Harlan Laboratories where rabbit immunizations and bleedings were done according to their standard protocols. Production bleeds with the highest concentration of DYNLT1 antibody as detected by immunoblotting on K562 cell lysate and purified DYNLT1 were selected for antibody purification. At each step of purification, serum and antibody fractions were tested by immunoblotting of K562 cell lysate (Fig. S1). Samples were subjected to SDS-PAGE and transferred to PVDF membranes. Membrane strips were prepared and incubated with dilutions of pre-bleed serum, test bleeds, production bleeds, samples from different stages of purification and collected fractions (Fig. S1). An HRP-conjugated anti-rabbit antibody was used as secondary antibody. Antiserum (Fig. S1, lane 1) was first precleared for 4 h at 4°C using 1 ml Affigel 15 beads (Bio-Rad) loaded with lysate containing 30 mg protein from *E. coli* BL21 bacteria expressing empty pGEX6P1 vector (Fig. S1, lane 2). The pre-cleared serum was added to a column containing 1 ml Affigel 15 beads loaded with 1 mg DYNLT1 protein and incubated overnight with rotation at 4°C. The flow-through fraction was depleted of DYNLT1 antibody as shown in Fig. S1, lane 3. The column was washed 6 times with 10 ml cold DPBS at 4°C. Bound antibody was eluted once with 4 ml 1 M NaCl in DPBS, once with 4 ml 0.1 M Glycine, pH 2.5 and once with 4 ml 0.1 M Glycine pH 2.5, 1 M NaCl, 1% CHAPS. For each elution, 0.5 ml fractions were collected, and glycine-containing fractions were neutralized with 25 µl of 1 M Tris-base. Antibody concentration in the various fractions was determined by Amido Black staining and immunoblotting of purified recombinant DYNLT1 or K562 lysate (Fig. S1, lanes 4–11). More than 90% of the antibody was detected in fractions 4, 5 and 6 of the 0.1 M Glycine pH 2.5 elution (Fig. S1, lanes 7–9); these fractions were stored at −80°C. Specificities of the antibodies were determined by immunobloting either purified recombinant proteins or K562 lysates. To further test the anti-DYNLT1 antibody, K562 cells were nucleofected with empty pRFP-C-RS vector, vector containing nonspecific shRNA, or vectors containing two different shRNAs against DYNLT1 (Origene) (see Table 1 for sequences). Lysates were subjected to SDS-PAGE and DYNLT1 was detected with the anti-DYNLT1 antibody.

**Table 1:** Non-specific and DYNLT1-specific shRNA/siRNA sequences.

Target	Company	shRNA Sequence
Non-Specific	Origene	GCACTACCAGAGCTAACTCAGATAGTACT
Human DYNLT1	Origene	GCTGGATTACACACAGCAAGTTCCTGCTT
Human DYNLT1	Origene	GGTGGTAACGCTTATCAACACAGCAAAGT
		**siRNA Sequence**
Non-specific	Invitrogen	Not provided
Human DYNLT1	Invitrogen	CAAAGTGAACCAGTGGACCACAAAT
Human DYNLT1	Invitrogen	GAACAAACTTTAAGCCAACTCACCA
Human DYNLT1	Invitrogen	TCAGTGCCTTCGGACTGTCTATTTG

### Immunofluorescence Microscopy

For localization of DYNLT1, 25,000 HEPG2 (ATCC) cells were grown overnight on poly-L- lysine coated coverslips (Neuvitro), fixed with methanol/1mM EGTA for 10 min at −20°C, washed with PBS twice, and blocked with 2% normal goat serum in DPBS with 1% BSA, 0.1% Tween 20. Primary antibodies used were rabbit anti-DYNLT1 along with mouse MAb 414 (Abcam). The fluorescent secondary antibodies used were FITC-conjugated anti-rabbit IgG from goat (Santa Cruz Biotechnology) and rhodamine-conjugated anti-mouse IgG from goat (Millipore Corp.). The slides were mounted with Vectashield mounting medium with DAPI (Vector Laboratories). The images were captured using an Eclipse 80i fluorescent microscope (Nikon) and processed in Metamorph ver 6.3r2 software (Molecular Devices).

### Co-immunoprecipitation

For DYNLT1 immunoprecipitation with NUP98-HOXA9, 10^7^ K562 cells were nucleofected with pcDNA-HA-NUP98-HOXA9 using a Nucleofector device (Lonza). The co-immunoprecipitations of NUP62 or NUP153 or NUP98 with DYNLT1 were done in untransfected K562 cells as described previously [16]. Briefly, cells were harvested 16 h post nucleofection, washed with cold DPBS, and lysed for 30 min on ice with 0.5 ml of lysis buffer (10 mM Tris pH 7.5, 0.4 M NaCl, 0.4% Triton X-100, 1% NP-40, 0.2% sodium deoxycholate, 1 mM EDTA, protease inhibitors (Roche), 1 mM PMSF). Dilution buffer (10 mM Tris pH 7.5, 1 mM EDTA, 1 mM PMSF, protease inhibitors) (0.5 ml) was added to the lysate, followed by centrifugation at 17,000×g for 30 min at 4°C. The supernatants were transferred to new tubes and either rabbit IgG or rabbit anti-DYNLT1 antibody was added. After overnight incubation at 4°C, 30 µl Protein G beads were added and incubated for another 1 h. Beads were washed with 700 µl of wash buffer (10 mM Tris pH 7.5, 1 mM EDTA, 0.2 M NaCl, 0.2% Triton X-100, 0.5% NP-40, 0.1% sodium deoxycholate, 1 mM PMSF, 1X protease inhibitors) 5 times, 3 min each time, followed by centrifugation at 1800×g for 3 min at 4°C. Beads were washed with cold DPBS and bound proteins were eluted by boiling with 30 µl of 2X SDS buffer (0.1 M Tris pH 6.8, 10% Glycerol, 3.5% SDS, 2 mM DTT, 0.004% Bromophenol Blue) for 10 min. Input lysates and immunoprecipitated proteins were subjected to SDS-PAGE (7.5% gel) followed by immunoblotting. NUP98-HOXA9 was detected with an anti-HA antibody; NUP98 with anti-NUP98 antibody (Cell Signaling Technology); NUP62 or NUP153 with mouse MAb414 antibody.

### Luciferase Assay

For analysis for DYNLT1 deletion mutants, K562 cells were nucleofected with 5 µg pGL4.11 vector driven by the *KBTBD10* promoter and 10 µg pcDNA-HA-NUP98-HOXA9 in combination with 10 µg of empty pcDNA3, pcDNA-DYNLT1, pcDNA-DYNLT1 N-terminus, or pcDNA-DYNLT1 C-terminus. For shRNA-mediated knockdown of DYNLT1, K562 cells were nucleofected with 5 µg pGL4.11 vector driven by the *KBTBD10* promoter in combination with 10 µg of either empty pcDNA3, or pcDNA3 expressing HA-NUP98-HOXA9. In addition, either empty pRFP-C-RS vector (Origene-TR30014), vector containing non-specific shRNA (Origene-TR30015), or vectors containing two different shRNA against DYNLT1 (Origene-TF313329) were included (Table 1). Knockdown of DYNLT1 was confirmed by immunoblotting with the anti-DYNLT1 antibody and the efficiency of knockdown was measured by densitometric analysis (Fig. S2). To control for efficiency of transfection, 0.5 µg of pRL-TK (Promega), which expresses Renilla luciferase was included. Five million cells were incubated with the DNA at room temperature for 10 min before electroporation and 10 min after electroporation, and were cultured in 10 ml IMDM media with 10% FBS, 2 mM L-glutamine, and 100 units/ml penicillin/streptomycin. Luciferase activity was measured 48 h after electroporation using the Dual Luciferase Reporter Assay System (Promega) and the results were normalized to Renilla luciferase.

### Retroviral Transduction and Primary Human CD34+ Cells Proliferation Assay

Frozen human CD34+ cells from healthy volunteers (Fred Hutchinson Cancer Research Center) were prestimulated and transduced with MSCV-IRES-YFP retrovirus expressing HA-NUP98-HOXA9 as previously described [17]. After 48 h, YFP positive cells were isolated using a MoFlo high-speed sorter (Dako) and expression of the NUP98-HOXA9 was confirmed by immunoblotting with anti-HOXA9 antibody. For DYNLT1 knockdown, NUP98-HOXA9 transfected CD34+ cells were grown for two weeks and nucleofected with either non-specific siRNA (Invitrogen-12935-300) or siRNA specific for DYNLT1 (Invitrogen-1299003) (Table 1). An MTS cell proliferation assay was done using CellTiter 96® AQueous NonRadioactive Cell Proliferation Assay (Promega G5421) according to the manufacturer's instructions. Twenty five thousand transfected cells were seeded into a 96-well plate in triplicate and grown for 24 h, 48 h and 72 h in 100 µl of culture medium. Twenty µl of MTS/phenazine methosulfate solution was added and the cells were incubated at 37°C for 2 h. The absorbance at 490 nm was measured using an ELISA plate reader. Knockdown of DYNLT1 was confirmed by immunoblotting using the anti-DYNLT1 antibody.

## Results

### Cytotrap Two-hybrid Analysis shows that NUP98-HOXA9 Interacts with Dynein Light Chain 1 (DYNLT1)

We used a Cytotrap yeast two-hybrid assay to identify possible interaction partners of the oncoprotein NUP98-HOXA9. The traditional two-hybrid assay is based on intranuclear interactions and is likely to show many false positives when the bait contains a transactivation domain such as the N-terminus of NUP98-HOXA9 [13]. In the Cytotrap yeast two-hybrid assay, the cDNA library expressing the target genes is subcloned downstream of a myristylation signal that localizes the proteins to the plasma membrane and the bait protein is fused to hSOS which remains in the cytoplasm. A physical interaction between the bait protein and one of the cDNA targets, results in recruitment of the hSOS protein to the plasma membrane, which causes activation of the Ras signaling pathway. This allows the cdc25H strain to grow and form colonies at the restrictive temperature (37°C) in galactose-containing medium. The bait protein was prepared by fusing NUP98-HOXA9 to hSOS and the target cDNA library was prepared from human K562 cells and subcloned into the pMYR vector. Expression of hSOS-NUP98-HOXA9 in the cdc25H yeast strain was confirmed by immunoblotting [16]. Controls for the assay were performed according to manufacturer’s instructions as previously described [16]. The cdc25H heat-sensitive yeast strain was transformed with both the bait protein and the target cDNA library. Several hundred colonies appeared and were replica-plated onto galactose-containing medium to induce expression of the target library. Colonies growing on the galactose-containing medium were isolated and selection in stringent temperature and media was repeated for selection of true positive clones. The cDNA inserts from the positive clones were isolated and sequenced. Several of the positive clones encoded full length Dynein Light Chain 1 (DYNLT1).

**Figure 1:**
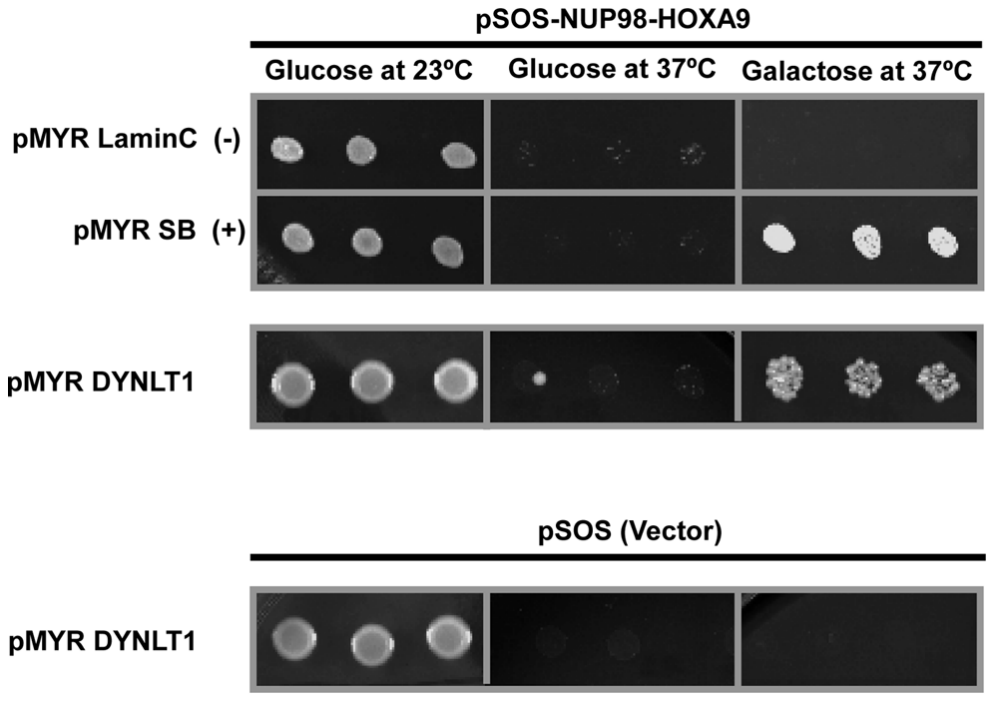
Cytoplasmic yeast two-hybrid analysis identifies DYNLT1 as an interacting partner for NUP98-HOXA9. K562 cDNA library in pMyr vector was co-transformed with pSOS-NUP98-HOXA9 into cdc25H yeast strain. Isolated colonies were patched on galactose-containing medium and transferred to 37°C. Patches growing at 37°C were re-spotted on plates containing selective medium supplemented with either glucose (repressing) or galactose (de-repressing) as sole carbon source and incubated either at 23°C (permissive) or 37°C (restrictive) for 3–5 days. Plasmids were isolated from yeast cells growing at 37°C in galactose containing medium and retransformed along with pSOS-NUP98-HOXA9 to confirm the interactions. Several positive cDNA clones were sequenced and identified as DYNLT1. Cells containing pSOS-NUP98-HOXA9 with pMYR-LaminC or empty pSOS vector with pMyr-DYNLT1 were spotted as negative controls. Cells containing pSOS-NUP98-HOXA9 with pMYR-SB (SOS Binding protein) were spotted as a positive control. Growth at 37°C in galactose containing medium indicates a positive interaction.

To confirm the interaction between NUP98-HOXA9 and DYNLT1, the plasmids encoding DYNLT1 along with either pSOS vector or vector expressing hSOS-NUP98-HOXA9 were reintroduced into the cdc25H yeast strain. Colonies were isolated and patched into positive selective medium and subjected to the restrictive temperature. Co-transfection of with the pSOS-NUP98-HOXA9 bait construct along with a plasmid expressing myristylated Lamin C into cdc25 strain served as the negative control. Cells co-transfected with the pSOS-NUP98-HOXA9 bait construct and a plasmid expressing myristylated SOS-binding protein (SB) served as the positive control. As shown in Fig. 1 growth was observed in all transformants incubated at 23°C. No growth was observed at 37°C in glucose containing medium because the cDNA library is cloned under a galactose-inducible promoter and was not expressed in the absence of galactose. When transferred to restrictive 37°C, no growth was seen in cells co-expressing pMYR-LaminC and pSOS-NUP98-HOXA9 indicating that NUP98-HOXA9 does not activate Ras signaling by targeting itself to the plasma membrane. Growth was present in cells co-expressing myristylated SB (SOS binding protein) and pSOS-NUP98-HOXA9 because the membrane-anchored SB protein recruits hSOS-NUP98-HOXA9 to the plasma membrane by binding to the hSOS portion. Cells co-transfected with hSOS-NUP98-HOXA9 and DYNLT1 showed a similar growth pattern suggesting a specific interaction between DYNLT1 and NUP98-HOXA9. The positive interaction observed with hSOS-NUP98-HOXA9 is not due to binding of DYNLT1 to the hSOS portion of the bait as there was no growth in cells transfected with the empty hSOS vector and DYNLT1 (Fig. 1).

### DYNLT1 Interacts with the FG Repeat Region of NUP98-HOXA9

**Figure 2:**
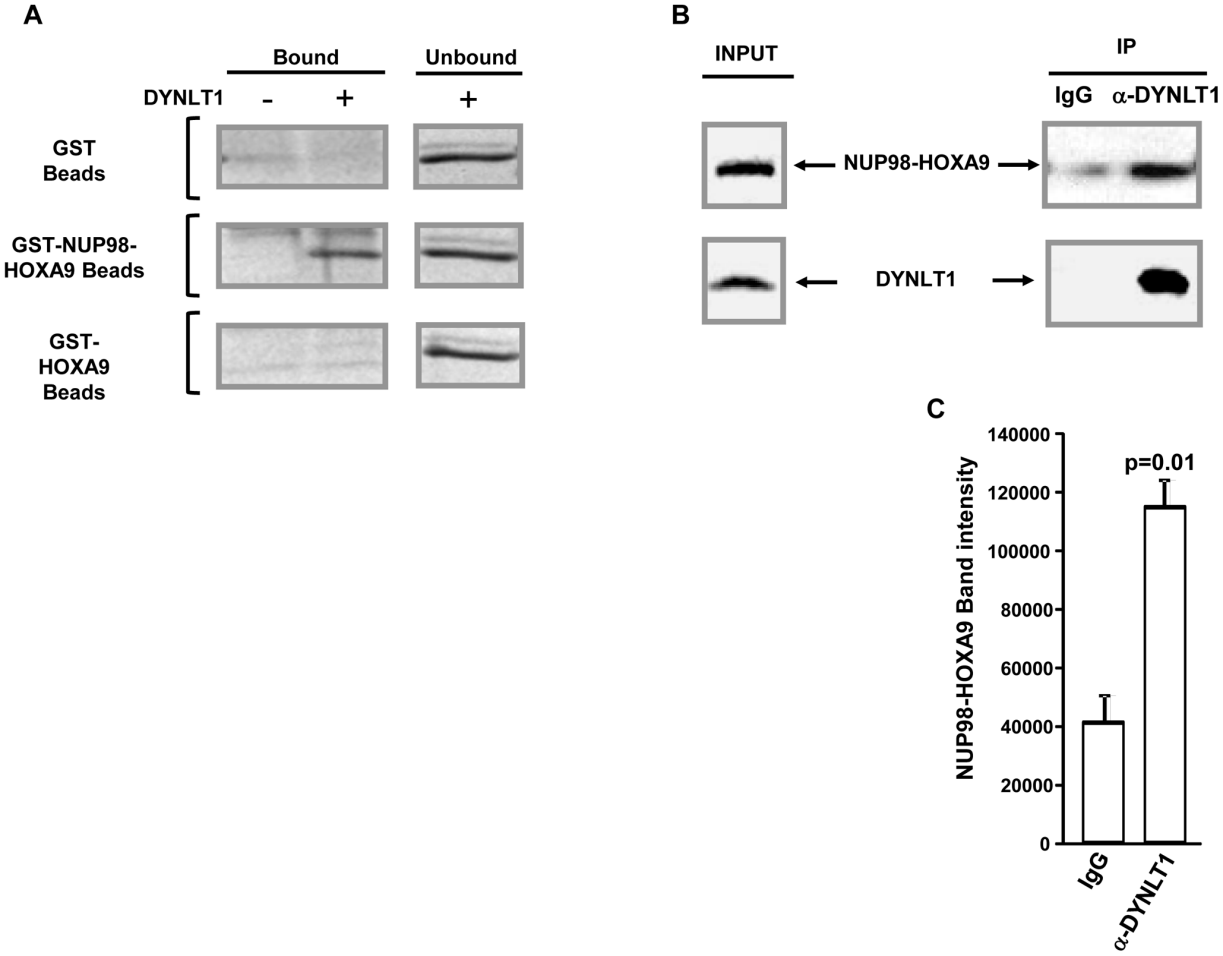
DYNLT1 interacts with NUP98-HOXA9*in vitro* and *in vivo*. *A)* GST, GST-NUP98-HOXA9, or GST-HOXA9 were immobilized on beads and incubated with purified recombinant DYNLT1 protein. The samples were subjected to SDS-PAGE and coomassie stained. *B)* Co-immunoprecipitation of NUP98-HOXA9 with DYNLT1 in K562 cells transfected with HA-NUP98-HOXA9. Whole cell lysates were subjected to immunoprecipitation with either rabbit IgG or anti-DYNLT1 antibody. NUP98-HOXA9 in the cell lysates (Input) and immunoprecipitated complexes (IP) was detected by immunoblotting with anti-HA antibody. DYNLT1 was detected by immunoblotting with anti-DYNLT1 antibody. *C)* Densitometric quantification of immunoprecipitated NUP98-HOXA9 with control IgG or anti-DYNLT1 antibody.

To establish a direct interaction between DYNLT1 and NUP98-HOXA9 an *in-vitro* binding assay was carried out using purified recombinant proteins. GST, GST-NUP98-HOXA9, or GST-HOXA9 were immobilized on beads and incubated with purified DYNLT1 protein. As shown in Fig. 2A, GST-NUP98-HOXA9 interacted with DYNLT1 whereas no interaction was observed for GST or GST-HOXA9. This suggests that DYNLT1 specifically interacts with the NUP98 portion of the NUP98-HOXA9 fusion protein.

To demonstrate that NUP98-HOXA9 interacts with DYNLT1 *in vivo*, co-immunoprecipitation was carried out using K562 cells transfected with HA-tagged NUP98-HOXA9. As the affinity and specificity of the commercially available DYNLT1 antibody were not optimal (data not shown), a rabbit polyclonal antibody was raised (Fig. S1) and used for immunoprecipitation. Whole cell lysates were subjected to immunoprecipitation with either rabbit IgG or anti-DYNLT1 antibody and probed for NUP98-HOXA9 with anti-HA antibody. As shown in Figs. 2B and 2C, NUP98-HOXA9 co-immunoprecipitated with endogenous DYNLT1, confirming their interaction within human myeloid cells.

**Figure 3:**
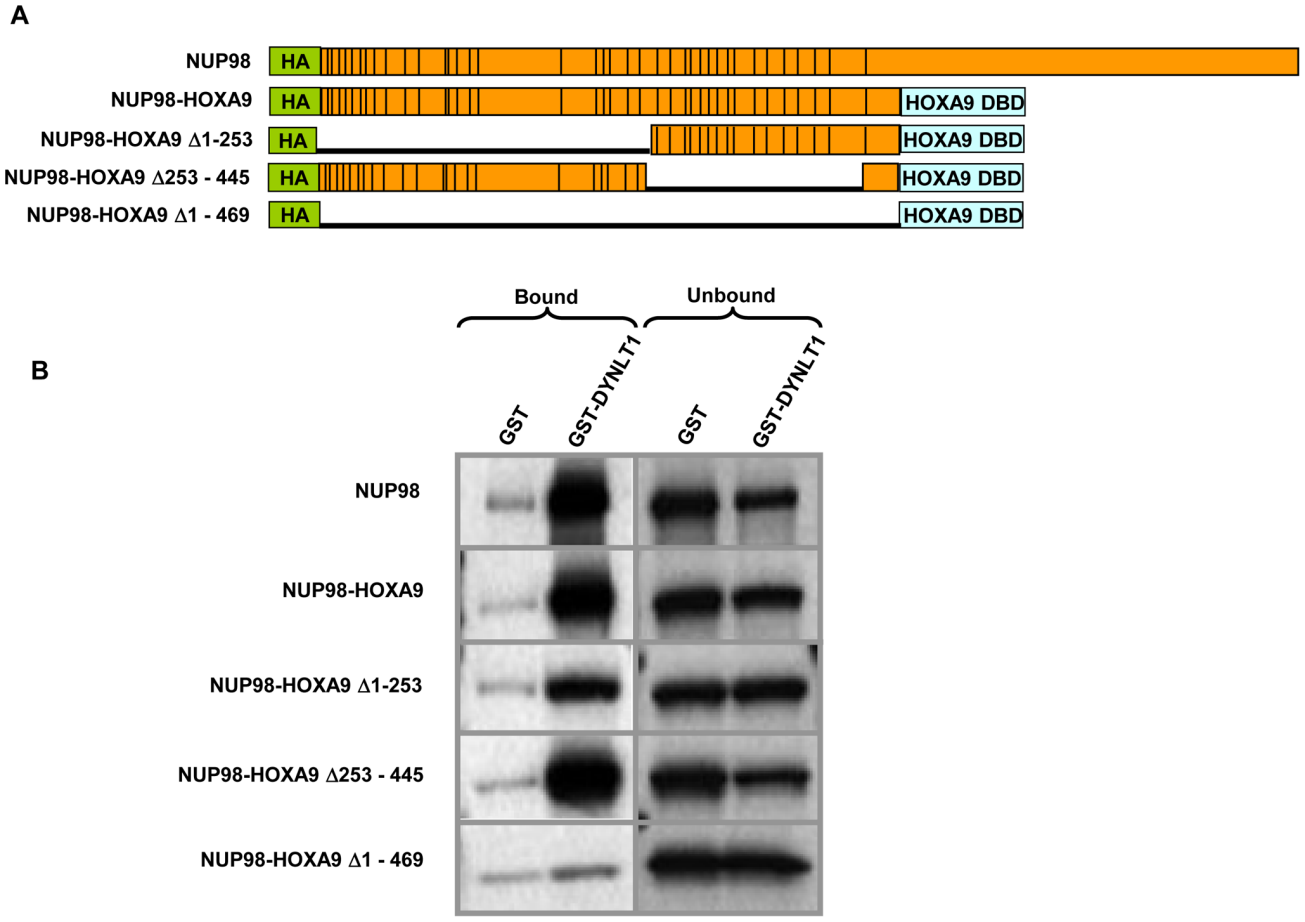
DYNLT1 interacts with the FG repeat region of NUP98-HOXA9. *A)* Full length NUP98, NUP98-HOXA9 and NUP98-HOXA9 deletion mutants. Vertical lines represent FG repeats. DBD = DNA-binding domain. *B)* GST or GST-DYNLT1 were immobilized on beads and incubated with *in vitro* translated full length NUP98, NUP98-HOXA9, or deletion mutants of NUP98-HOXA9. Bound and unbound fractions were resolved by SDS-PAGE and subjected to autoradiography.

To define essential regions in NUP98-HOXA9 responsible for the interaction with DYNLT1, GST-DYNLT1 was immobilized on beads and incubated with *in-vitro* translated full length NUP98, NUP98-HOXA9, or deletion mutants of the NUP98 portion of NUP98-HOXA9. The NUP98-HOXA9 mutants were deleted for the N-terminus (Δ1–253), C-terminus (Δ253–445) or the entire NUP98 portion (Δ1–469) (Fig. 3A). DYNLT1 interacted with full length NUP98 and NUP98-HOXA9 to a similar extent (Fig. 3B). Removal of the C-terminal portion of the NUP98 moiety (Δ253–445) did not affect binding of NUP98-HOXA9 to DYNLT1. On the other hand, when the N-terminal of NUP98-HOXA9 was deleted (Δ1–253), the interaction was significantly reduced. The interaction of DYNLT1 with NUP98-HOXA9 was abolished by deletion of the entire NUP98 moiety (Fig. 3B). These data show that DYNLT1 interacts with the FG repeat-rich NUP98 moiety of NUP98-HOXA9 and that the first 253 amino acids of NUP98-HOXA9 are required for optimal interaction with DYNLT1.

### DYNLT1 Colocalizes with the Nuclear Pore Complex

**Figure 4:**
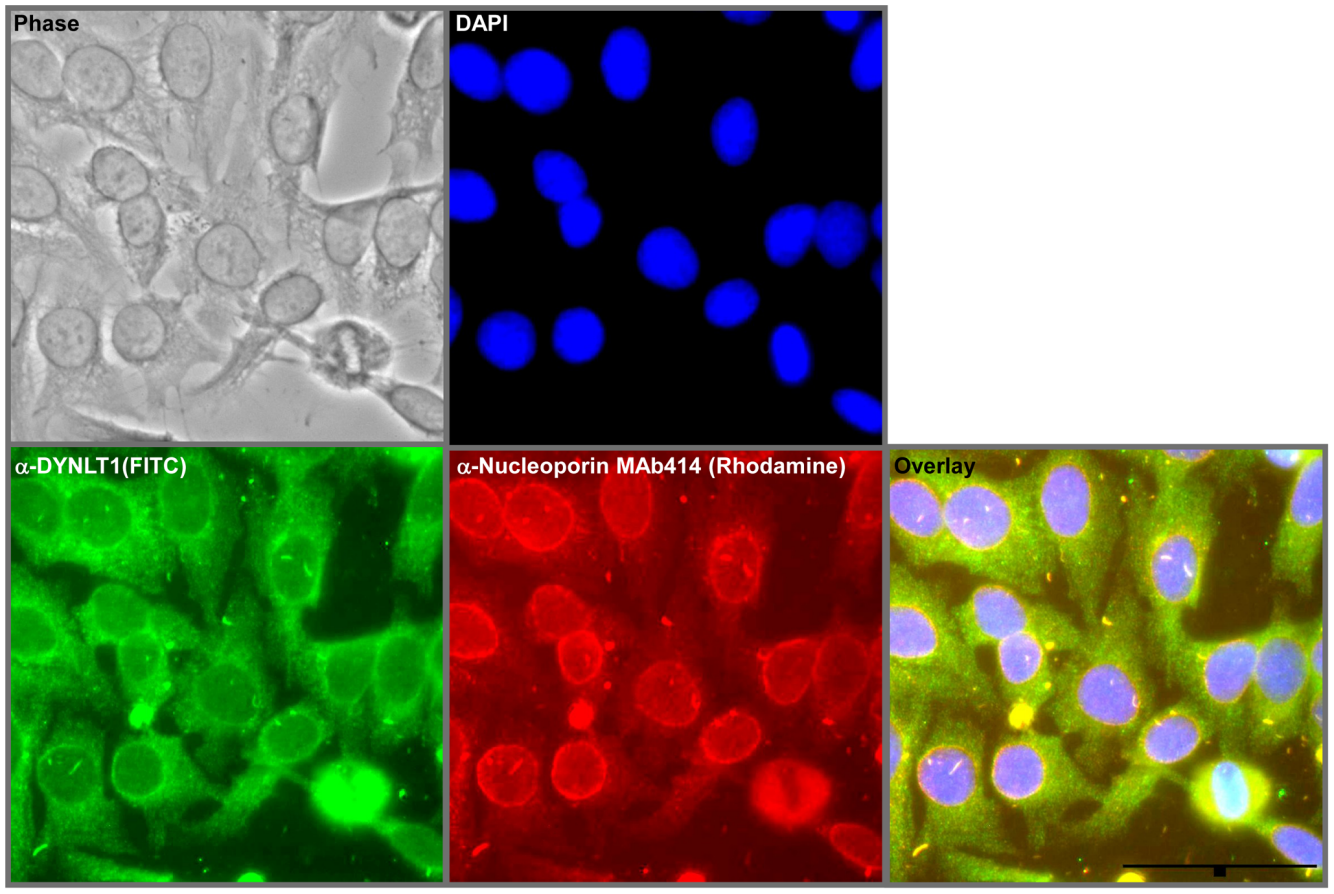
DYNLT1 localizes to the cytoplasm and the nuclear periphery. HEPG2 cells were grown on a coverslip and permeabilized with methanol. Cells were immunostained with the anti-DYNLT1 (green) antibody together with MAb414 (red) that marks the nuclear periphery by staining nucleoporins. The nuclei were counterstained with DAPI. Scale bar = 50 µm.

DYNLT1, also known as Tctex-1, is an integral 14 KDa protein subunit of the large microtubule-based cytoplasmic dynein complex [26], [27], [28]. The interaction of DYNLT1 with the FG repeat region of NUP98-HOXA9 suggests that DYNLT1 may interact with other FG repeat nucleoporins and localize to the nuclear pore complex. To determine the intracellular localization of DYNLT1 in relation to the nuclear pore complex, human HEPG2 cells were immunostained with rabbit anti-DYNLT1 and mouse MAb414 that highlights the nuclear pore complexes [30]. A FITC-conjugated anti-rabbit secondary antibody was used to detect DYNLT1 and a rhodamine-conjugated anti-mouse secondary antibody was used to highlight the nucleoporins. DYNLT1 was found throughout the cell and formed a ring around the nuclear periphery where it co-localized with the nuclear pore complexes (Fig. 4).

### DYNLT1 Interacts with FG Repeat Nucleoporins

**Figure 5:**
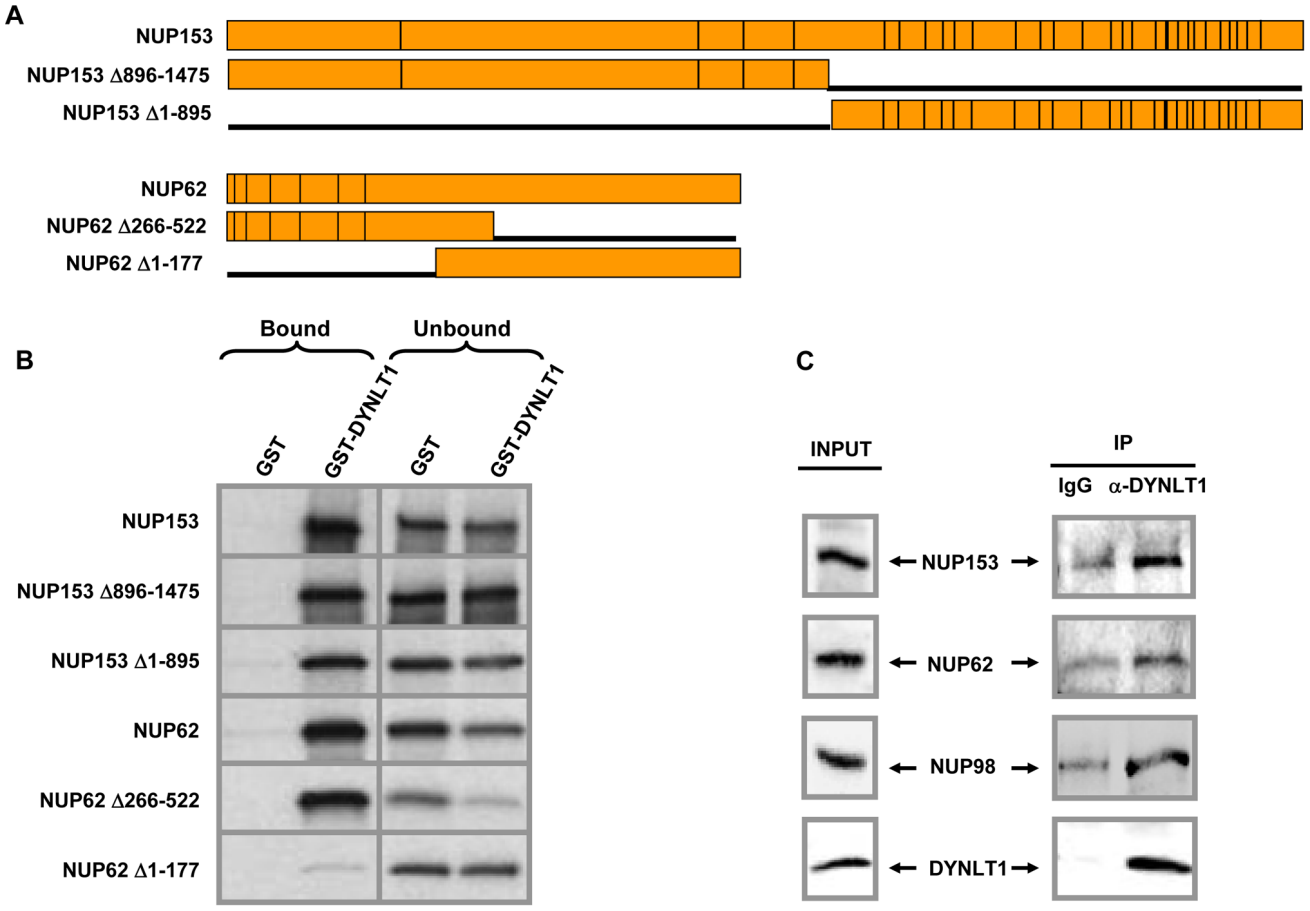
DYNLT1 interacts with FG repeat regions of NUP153 and NUP62. *A)* Full length NUP153, NUP153 deletion mutants, NUP62 and NUP62 deletion mutants. Vertical lines represent FG repeats. *B)* GST or GST-DYNLT1 immobilized on beads were incubated with *in vitro* translated NUP153, NUP62, or their deletion mutants. Bound and unbound fractions were resolved by SDS-PAGE and subjected to autoradiography. *C)* Co-immunoprecipitation of NUP153, NUP62 and NUP98 with DYNLT1 in K562 cells. Whole cell lysates were subjected to immunoprecipitation with either rabbit IgG or anti-DYNLT1 antibody. Both input and immunoprecipitates (IP) were probed for NUP153 or NUP62 using the MAb414 antibody or NUP98 using anti-NUP98 antibody. DYNLT1 was detected with anti-DYNLT1 antibody.

The immunolocalization of DYNLT1 to the nuclear rim and its binding to the FG repeat portion of NUP98 raised the possibility that DYNLT1 interacts with other FG repeat nucleoporins. To determine whether this is the case, GST-DYNLT1 immobilized on beads was incubated with *in vitro* translated NUP153, NUP62, or their deletion mutants (Fig. 5A). As shown in Fig. 5B, DYNLT1 interacts with both NUP153 and NUP62. The binding of DYNLT1 to NUP153 was mildly diminished by deletion of either the N- or C-terminus. On the other hand, removal of the FG-repeat containing N-terminus of NUP62 almost completely abolished the interaction with DYNLT1.

To confirm that DYNLT1 interacts with nucleoporins *in vivo*, K562 cell lysates were subjected to immunoprecipitation with either rabbit IgG or anti-DYNLT1 antibody and probed for NUP153 and NUP62 using the mouse MAb414 antibody and for NUP98 using the rabbit anti-NUP98 antibody. As shown in Fig. 5C, DYNLT1 coimmunoprecipitates with NUP153, NUP62 and NUP98.

**Figure 6:**
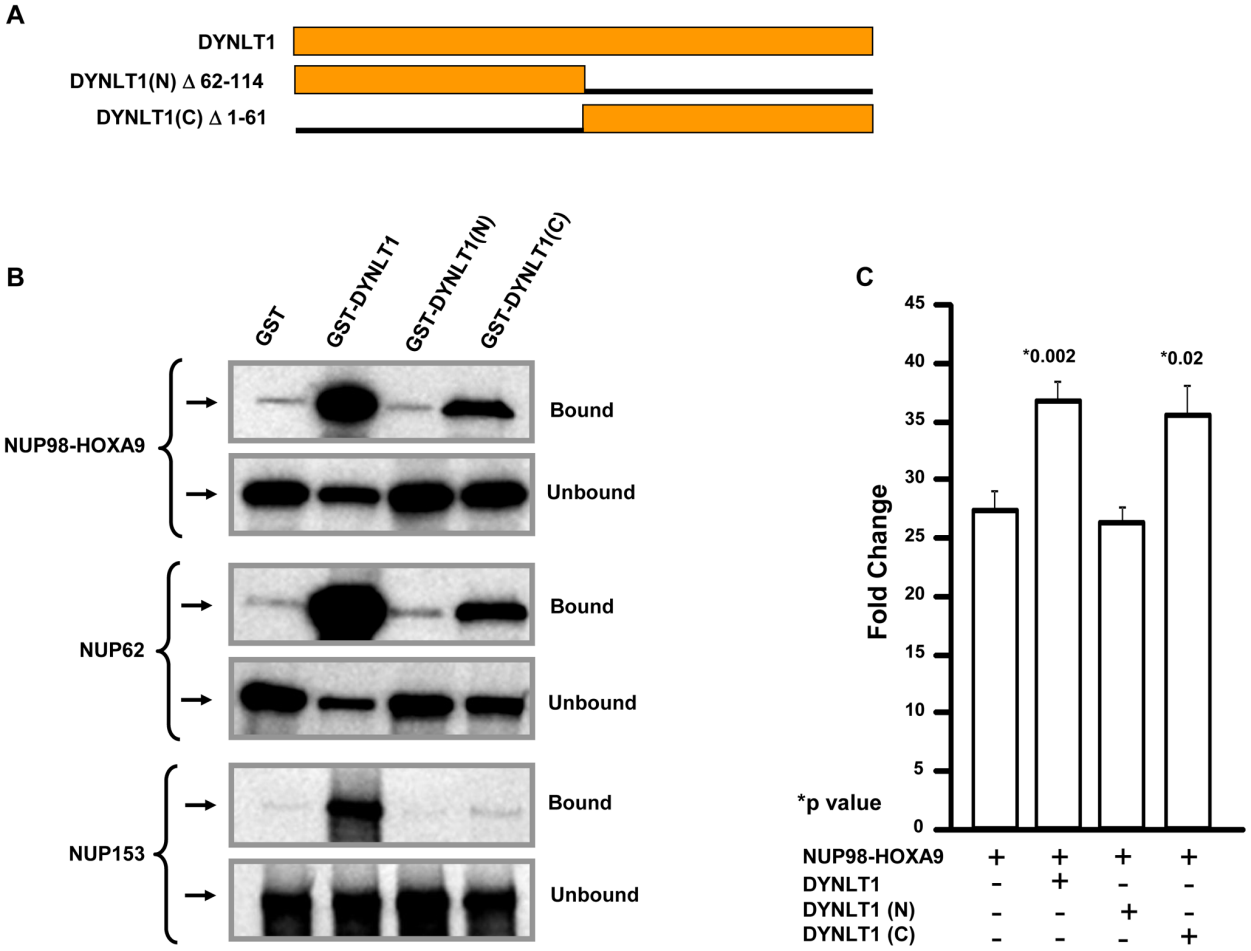
C-terminus of DYNLT1 interacts with NUP98-HOXA9, NUP62 and NUP153. *A)* Full length DYNLT1 and deletion mutants. *B*) GST or GST-DYNLT1 or GST-DYNLT1 N-terminus or GST-DYNLT1 C-terminus with *in vitro* translated NUP98-HOXA9, NUP62 or NUP153. Bound and unbound fractions were resolved by SDS-PAGE and subjected to autoradiography. *C)* K562 cells were nucleofected with pGL4.11 vector driven by the *KBTBD10* promoter and pcDNA-HA-NUP98-HOXA9 in combination with either empty pcDNA3, pcDNA-DYNLT1, pcDNA-DYNLT1 N-terminus, or pcDNA-DYNLT1 C-terminus. Firefly luciferase activity was measured 48 hours after transfection and normalized to a Renilla luciferase internal control. The numbers represent fold change over control (average of 3 independent experiments); error bars represent standard deviations. The P values indicated were obtained by a two-tailed t-test in comparison to NUP98-HOXA9 alone.

To identify DYNLT1 domains that interact with NUP98-HOXA9 and nucleoporins, either GST, or GST fused to DYNLT1, DYNLT1 N-terminus (aa 1–61), or DYNLT1 C-terminus (aa 62–113) (Fig. 6A) were immobilized on beads. Pull down assays were performed with *in-vitro* translated NUP98-HOXA9, NUP62, or NUP153. As shown in Fig. 6B, the C-terminus of DYNLT1 showed weak interactions with NUP98-HOXA9 as well as nucleoporins NUP62 and NUP153, whereas the N-terminus of DYNLT1 showed no such interactions. Both the N-terminus and the C-terminus were needed for optimal interactions with NUP98-HOXA9, NUP62, and NUP153.

To test whether the DYNLT1 C-terminus, that interacts with NUP98-HOXA9 plays a role in transcriptional dysregulation by NUP98-HOXA9, a luciferase reporter construct driven by the *KBTBD10* promoter was introduced into K562 cells along with a construct expressing NUP98-HOXA9 in combination with full length DYNLT1 or DYNLT1 N-terminus or C-terminus. Overexpression of the full length DYNLT1 or the DYNLT1 C-terminus resulted in a significant increase in transcriptional activation of the reporter construct by NUP98-HOXA9, whereas overexpression of the DYNLT1 N-terminus did not result in increased transcriptional activity by NUP98-HOXA9 (Fig. 6C).

### DYNLT1 is Required for Transcriptional Activation by NUP98-HOXA9

**Figure 7:**
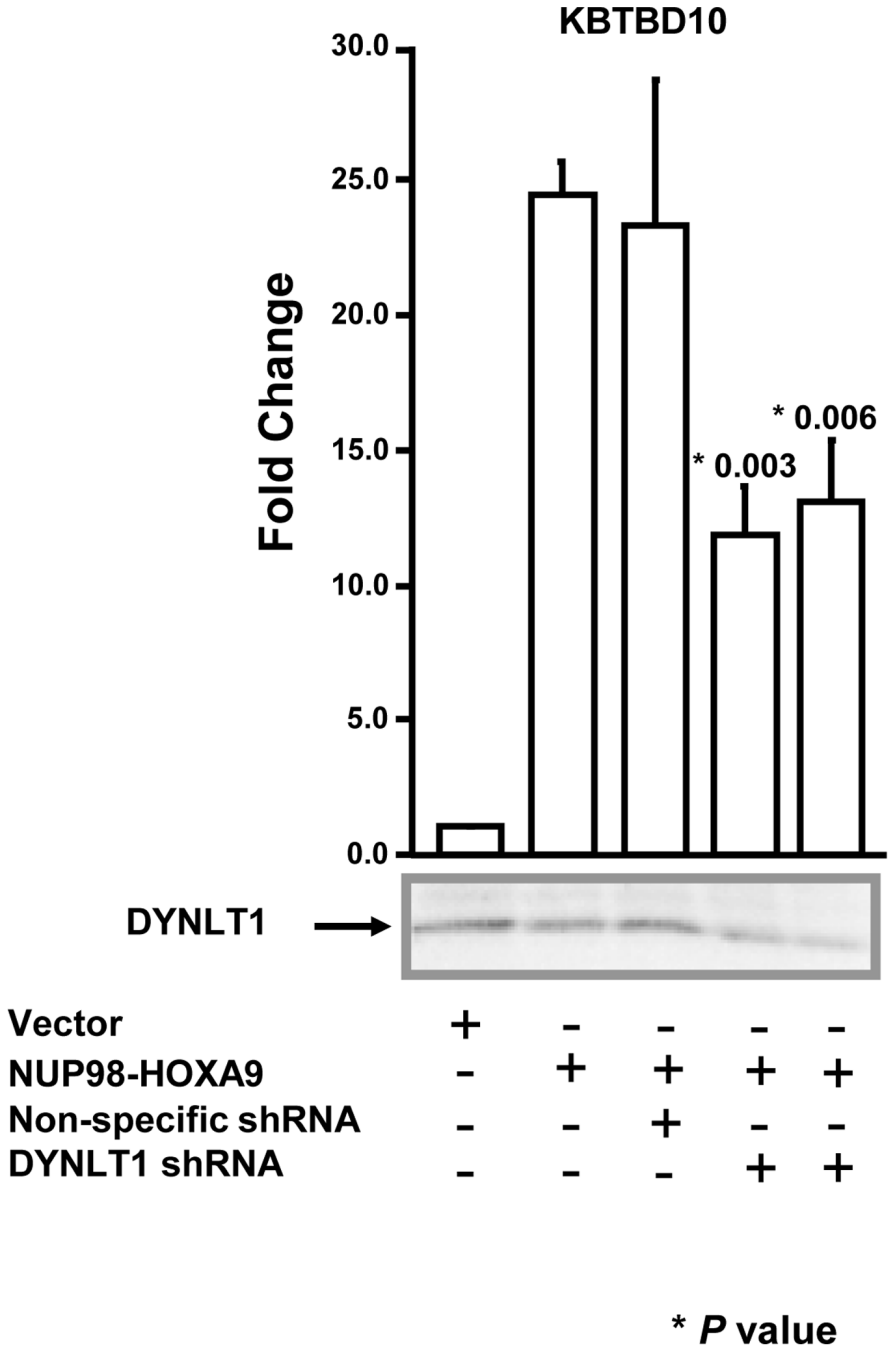
shRNA mediated knockdown of DYNLT1 inhibits downstream gene regulation by NUP98-HOXA9. K562 cells were transfected with a firefly luciferase construct driven by the *KBTBD10* promoter with either empty vector or vector expressing NUP98-HOXA9. In addition, the transfections included either empty vector, or vector expressing non-specific shRNA, or 2 vectors expressing different DYNLT1 shRNAs. Firefly luciferase activity was measured 48 hours after transfection and normalized to a Renilla luciferase internal control. The numbers represent fold change over control (average of 3 independent experiments); error bars represent standard deviations. The P value indicated was obtained by a two-tailed t-test. The knockdown of DYNLT1 were verified by immunoblotting with anti-HA or anti-DYNLT1 antibody.

NUP98-HOXA9 causes transcriptional dysregulation in human hematopoietic cells [12], [13], [17], [19]. In the human myeloid K562 cell line, we have previously identified the *KBTBD10* gene as a direct transcriptional target of NUP98-HOXA9 [19]. To test whether DYNLT1 plays a role in transcriptional dysregulation by NUP98-HOXA9, DYNLT1 was knocked down using two different anti-DYNLT1 shRNA constructs. A luciferase reporter construct driven by the *KBTBD10* promoter was introduced into K562 cells along with a construct expressing NUP98-HOXA9, with or without non-specific shRNA or one of the two DYNLT1-specific shRNA constructs. Knockdown of DYNLT1 was confirmed by immunoblotting (Figs. 7 and S2). Interestingly, knockdown of DYNLT1 by either of the shRNA constructs resulted in impairment of transcriptional activation of the *KBTBD10* promoter by NUP98-HOXA9 (Fig. 7). No such impairment of transcriptional activation was observed with the non-specific shRNA construct. This indicates that DYNLT1 plays a role in transcriptional dysregulation by NUP98-HOXA9 and raises the possibility that DYNLT1 plays a role in the leukemogenic effects of NUP98-HOXA9.

### Knockdown of DYNLT1 Inhibits NUP98-HOXA9 Mediated Proliferation of Primary Human CD34+ Cells

**Figure 8:**
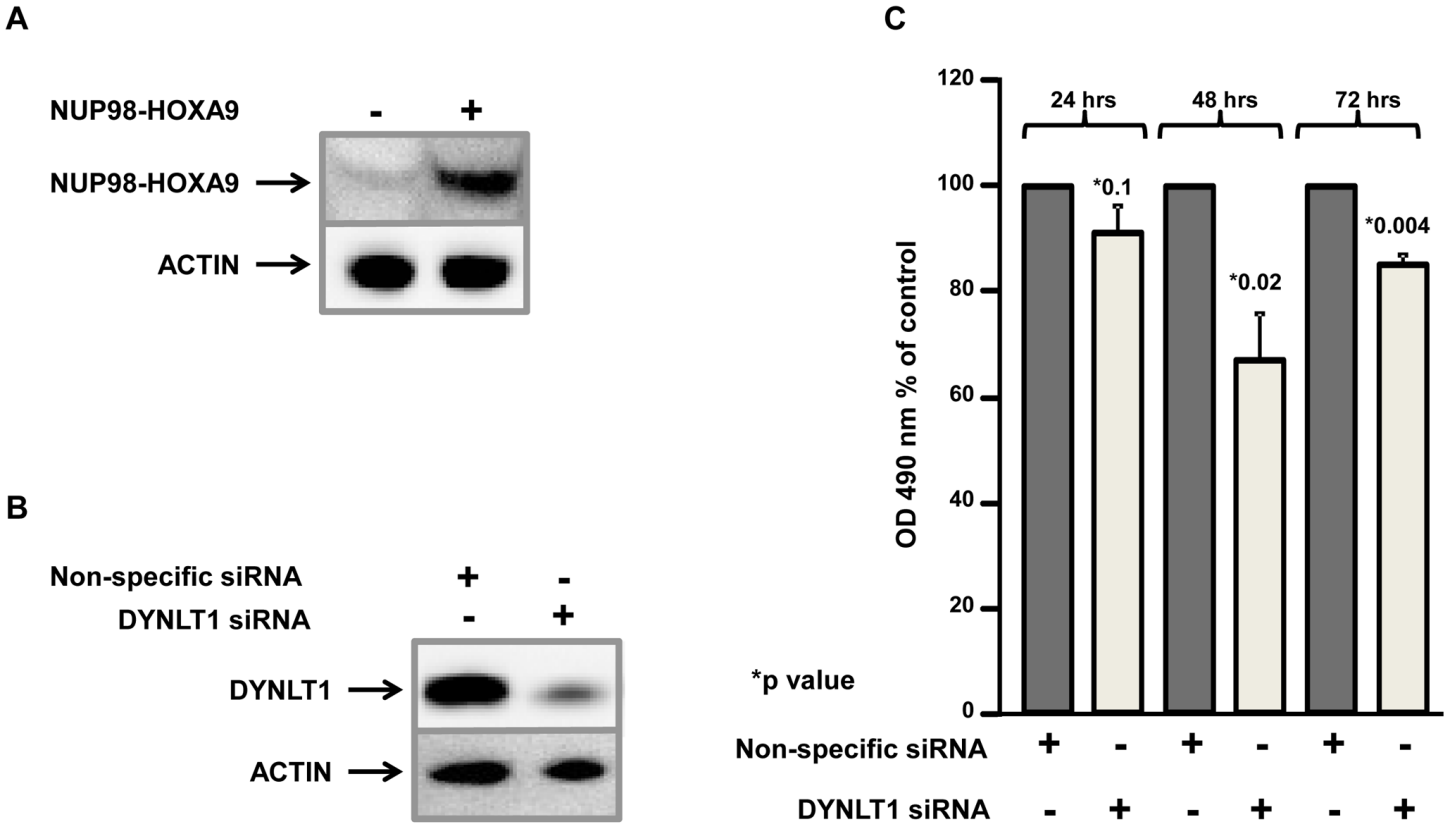
Knockdown of DYNLT1 inhibits proliferation of cells expressing NUP98-HOXA9. A) Primary human CD34+ cells were retrovirally transduced with either control MSCV-IRES-YFP vector or vector expressing NUP98-HOXA9. Cells positive for YFP were sorted and NUP98-HOXA9 was detected by immunoblotting with an anti-HOXA9 antibody; immunoblotting for actin was used as a loading control. B) Knockdown of DYNLT1 was confirmed by immunoblotting with the anti-DYNLT1 antibody; actin immunoblotting was used as a loading control.C) Sorted NUP98-HOXA9-expressing human primary CD34+ cells were transfected by nucleofection with non-specific siRNA and siRNA specific for DYNLT1. Cells were seeded into a 96-well plate in triplicate and cell proliferation was measured 24 h, 48 h and 72 h later by an MTS assay using CellTiter 96® AQueous Nonradioactive Cell Proliferation Assay. The y axis represents cell proliferation as measured by the absorbance at 490 nm. The error bars represent standard deviation from three independent experiments; the p-values were calculated using a two-tailed t-test.

To demonstrate the role of DYNLT1 in NUP98-HOXA9-mediated aberrant cell proliferation, human CD34+ primary cells were retrovirally transduced to express NUP98-HOXA9 from an MSCV-IRES-YFP vector. Transduced cells were isolated by sorting for YFP positivity, and expression of NUP98-HOXA9 was confirmed by immunoblotting (Fig. 8A). CD34+ cells expressing NUP98-HOXA9 were transfected with either nonspecific siRNA or DYNLT1 siRNA and were grown for 24 h, 48 h and 72 h and knockdown of DYNLT1 was confirmed by immunoblotting with anti-DYNLT1 antibody (Fig. 8B). An MTS cell proliferation assay was performed and showed that knockdown of DYNLT1 resulted in a significant inhibition of cell proliferation at 48 h and 72 h (Fig. 8C). These data suggest that DYNLT1 plays a role in the transformation of human hematopoietic cells by NUP98-HOXA9.

## Discussion

DYNLT1 is an integral 14 KDa protein subunit of the large microtubule-based cytoplasmic dynein complex [26], [27], [28]. There are four major subunits of this complex namely, the ∼530 KDa heavy chains (HC), ∼74 KDa intermediate chains (IC), ∼33–59 KDa intermediate light chains (ILC) and the ∼10–14 KDa light chains (LC) [31], [32], [33]. Three families of Dynein light chains have been identified: the t-complex–associated family (DYNLT1, DYNLT3), the Roadblock family (DYNLRB1, DYNLRB2), and the LC8 family (DYNLL1, DYNLL2) [33]. The two DYNLT1 polypeptides within the cytoplasmic dynein complex form homodimers and also interact with the N-terminus of the intermediate chain DYNC1I1 [34], [35], [36]. The dynein complex is involved in a variety of cellular functions including transport of membranous organelles, mitotic spindle orientation, nuclear migration, and cell migration [37]. Dynactin is part of an actin-related cytoplasmic protein complex that interacts with Dynein and is essential for its function [38], [39], [40].

In yeast, dynein light chain (Dyn2) localizes to the nuclear pore complex through its interaction with the FG repeat-containing nucleoporin Nup159, and it has been suggested that Dyn2 may play a role in organizing the natively unfolded FG repeats within the nuclear pore complex and promoting oligomerization of nucleoporin subcomplexes [41], [42]. There is evidence that ubiquitylation of NUP159 is necessary for recruitment of Dyn2 to the nuclear pore complex and this interaction facilitates the migration of nuclei to daughter cells during mitosis [43]. Homology search using the Saccharomyces Genome Database (SGD) http://www.yeastgenome.org/showed that yeast Dyn2 protein shares homology with both human DYNLL1 (50%) and DYNLL2 (47%) but not with DYNLT1.

In higher eukaryotes, several reports have shown evidence for an association between dynein and the nuclear envelope and/or the nuclear pore complex. These associations mostly occur during mitosis and are generally indirect. For example, in mammalian cells dynein intermediate chains have been localized to the nuclear envelope during prophase [44], [45]. Similarly, in Drosophila, dynein mediates the attachment of centrosomes to the nuclear envelope during mitosis [46]. Payne et al. showed that several nucleoporins coimmunoprecipitate with dynactin in the mammalian zygote; they suggested that the interaction between nucleoporins and dynactin is indirect and is mediated by vimentin [47]. It has also been shown in mammalian cells that dynein is recruited to the nuclear pore complex during the G2 phase through an interaction between the adaptor molecule, BICD2 and the nucleoporin RANBP2 (NUP358) [48]. Dynein light chain DYNLL1 and dynactin have also been shown to co-immunoprecipitate with the nucleoporin TPR in HeLa cells during mitosis [49]. The interaction with dynein was confirmed by pulldown assays using TPR prepared in a reticulocyte lysate system; however since the latter contains cellular proteins, it remains unclear whether the interaction is direct. A recent study has shown that during prophase the nucleoporin NUP133 anchors the dynein/dynactin complex to the nuclear envelope indirectly through interactions with CENP-F and NudE/EL [50]. Thus it appears that dynein interacts transiently and indirectly with components of the nuclear pore complex to facilitate cell division.

In contrast, the data presented here demonstrate, for the first time to our knowledge, that a cytoplasmic dynein component directly interacts with nucleoporins in mammalian cells and that the interaction occurs in interphase. We have shown that DYNLT1 co-localizes with the nuclear pore complex and binds to the nucleoporins NUP98, NUP62 and NUP153 as well as to the oncogenic nucleoporin fusion NUP98-HOXA9. All of these DYNLT1-interacting nucleoporins contain the highly conserved FG repeat regions [1]. In the case of NUP62 and NUP98-HOXA9 these FG repeat regions are required for binding to DYNLT1 (Figs. 3 and 5).

The function of DYNLT1 at the nuclear pore complex remains to be determined. One possibility is that it may have a function in nucleocytoplasmic transport. The nuclear localization of some viral and cellular proteins is facilitated by the cytoskeleton, particularly microtubules, by mediating their perinuclear accumulation [51]. For example, Roth et al. found evidence for the involvement of microtubules in the nuclear import of some proteins, such as the cancer-related proteins p53, pRB, and PTHrP, but not others [52], [53]. Similarly, microtubules play a role in the nuclear import of STAT1 and STAT5B [54], [55]. There is substantial evidence that the role of microtubules in nucleocytoplasmic trafficking of cellular proteins is mediated by dynein; examples include the nuclear import of the mineralocorticoid receptor, STAT5B, and NFκB [55], [56], [57], [58]. The role of the cytoskeleton and dynein in the nucleocytoplasmic transport of viral components is also very well documented, and different viral proteins interact with different dynein subunits, including DYNLT1 [59], [60], [61], [62].

DYNLT1 is also implicated in functions independent of the cytoplasmic dynein complex [63]. DYNLT1 was shown to mediate neuritogenesis during hippocampal neuronal development in a dynein-independent manner [64]. It was also identified as an activator of G-protein signaling in association with several receptor proteins like the Trk neurotrophin receptor, bone morphogenetic receptor type II and the parathyroid hormone receptors [65], [66], [67], [68]. DYNLT1 also interacts with Lfc, a guanine nucleotide exchange factor in a phosphorylation dependent manner to regulate neurogenesis [69], [70]. RP3, a dynein light chain subunit, localizes to the nucleus and is involved in transcriptional regulation [71]. Localization studies showed a sub-population of DYNLT1 in the nucleus [63]. We have also observed a population of nuclear DYNLT1 (Fig. 4) and have shown that DYNLT1 plays a role in transcriptional dysregulation by NUP98-HOXA9 (Fig. 7).

Structural analysis showed that each DYNLT1 core contains two α-helices followed by four β-strands [35]. We have separated the α-helix-containing regions form the β-strand-containing regions and found that functional interactions with NUP98-HOXA9 or nucleoporins occurred through the β-strand region (Fig. 6). The interactions between DYNLT1 and other components of the dynein complex also occur through this β-stand containing region, suggesting that the interactions of DYNLT1 with nucleoporins and with components of the dynein complex may be mutually exclusive [36]. Thus it is possible that DYNLT1 functions within the nuclear pore complex independent of the dynein complex. In yeast dynein light chains are integrated into the nuclear pore complex and provide structural support to the nuclear pore complex components [41], [42]. DYNLT1 may play a similar role in the mammalian nuclear pore complex.

Our data suggest a role for DYNLT1 in leukemogenesis. NUP98-HOXA9 is a leukemogenic oncoprotein that acts as an aberrant transcription factor [12], [13], [17], [19]. The N-terminal FG repeat region of NUP98-HOXA9 has been implicated in the ability of NUP98-HOXA9 to dysregulate transcription and to cause leukemic transformation [12], [13], [14], [19]. Here we have shown that DYNLT1 interacts with this FG repeat region and that depletion of DYNLT1 impairs the ability of NUP98-HOXA9 to dysregulate transcription and to induce proliferation of primary human CD34+ cells (Figs. 7 and 8). These data suggest that DYNLT1 cooperates with NUP98-HOXA9 in leukemogenesis. Indeed, a study in a leukemia mouse model showed that the axonemal dynein light chain 4 (Dnalc4) and dynactin 3 (Dctn3) cooperate with NUP98-HOXA9 in mediating leukemogenesis [72]. Dynein components have also been implicated in the pathogenesis of other tumors. For example, upregulation of DYNLL1 was shown to promote cell proliferation and tumorigenesis by stimulating cyclin-dependent kinase 2 activity in hormone-responsive cancers [73], [74]. Similarly, dynein light chain DYNLRB1 is upregulated in patients with hepatocellular carcinoma [75].

To summarize, we have identified DYNLT1 as a component of the nuclear pore complex and a novel interacting partner for the oncoprotein NUP98-HOXA9 that cooperates with it in transcriptional deregulation and in the induction of proliferation of primary human CD34+ hematopoietic precursors. These data establish a direct link between dynein and the nuclear pore complex and suggest a role for DYNLT1 in the pathogenesis of AML caused by NUP98 fusions. Further studies are needed to determine the function of DYNLT1 at the nuclear pore complex, its role in nucleocytoplasmic transport, and the mechanisms by which it contributes to transcriptional dysregulation by oncogenic NUP98 fusions.

## Supporting Information

Figure S1 **Purification of Rabbit anti-DYNLT1 antibody from serum.** K562 cell lysates were subjected to SDS-PAGE and transferred to Hybond C-extra nitrocellulose membrane. Membrane strips were subjected to immunoblotting with 1∶2000 dilutions of the indicated serum fractions and eluates. Lane 1: anti –DYNLT1 Rabbit Serum. Lane 2: anti –DYNLT1 Rabbit Serum/Bacterial lysate Flow through. Lane 3. Anti –DYNLT1 Rabbit Serum/DYNLT1 (N)-Affigel Beads Flow through. Lanes 4–11: Purified fractions 1–8 (0.1 M Glycine).
(TIF)

Figure S2 **Densitometric quantification of shRNA-mediated DYNLT1 knockdown.** K562 cells were transfected with a firefly luciferase construct driven by the *KBTBD10* promoter with either empty vector or vector expressing NUP98-HOXA9. In addition, the transfections included either empty vector, or vector expressing non-specific shRNA, or vectors expressing 2 different DYNLT1 shRNAs. The intensities of the DYNLT1 bands in Fig. 7 were quantified using Chemidoc Quantity One software (BioRad). The DYNLT1 band intensities are shown as % of the empty vector control in lane 1.
(TIF)
