# Supplementary figures and images for: Dynein Light Chain 1 (DYNLT1) Interacts with Normal and Oncogenic Nucleoporins
Source: PLoS One. 2013 Jun 26;8(6):e67032. doi: 10.1371/journal.pone.0067032 (PMC3694108; doi:10.1371/journal.pone.0067032)

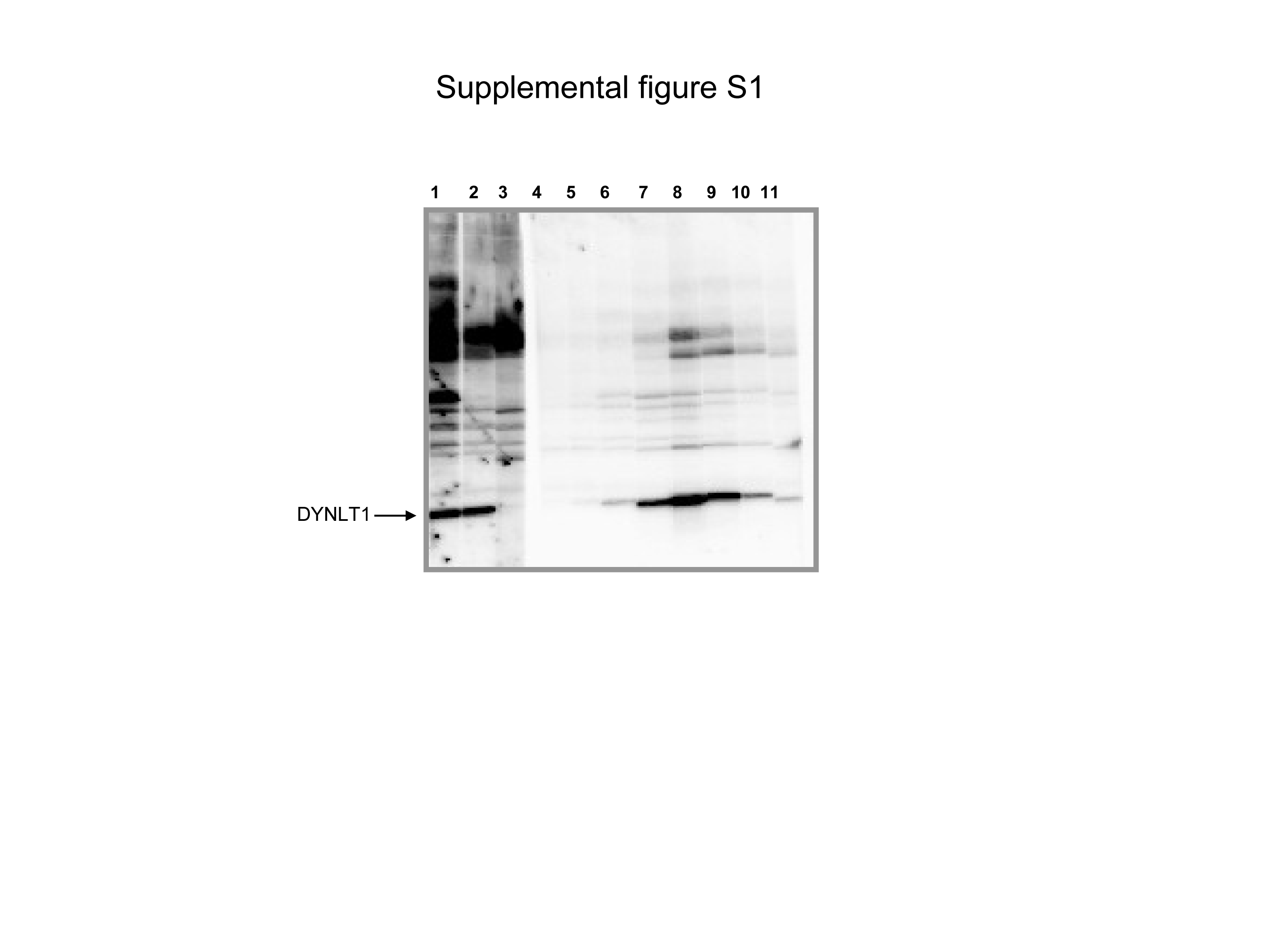

Supplement: Figure S1 — Purification of Rabbit anti-DYNLT1 antibody from serum. K562 cell lysates were subjected to SDS-PAGE and transferred to Hybond C-extra nitrocellulose membrane. Membrane strips were subjected to immunoblotting with 1∶2000 dilutions of the indicated serum fractions and eluates. Lane 1: anti –DYNLT1 Rabbit Serum. Lane 2: anti –DYNLT1 Rabbit Serum/Bacterial lysate Flow through. Lane 3. Anti –DYNLT1 Rabbit Serum/DYNLT1 (N)-Affigel Beads Flow through. Lanes 4–11: Purified fractions 1–8 (0.1 M Glycine). (TIF) [file pone.0067032.s001.tif]

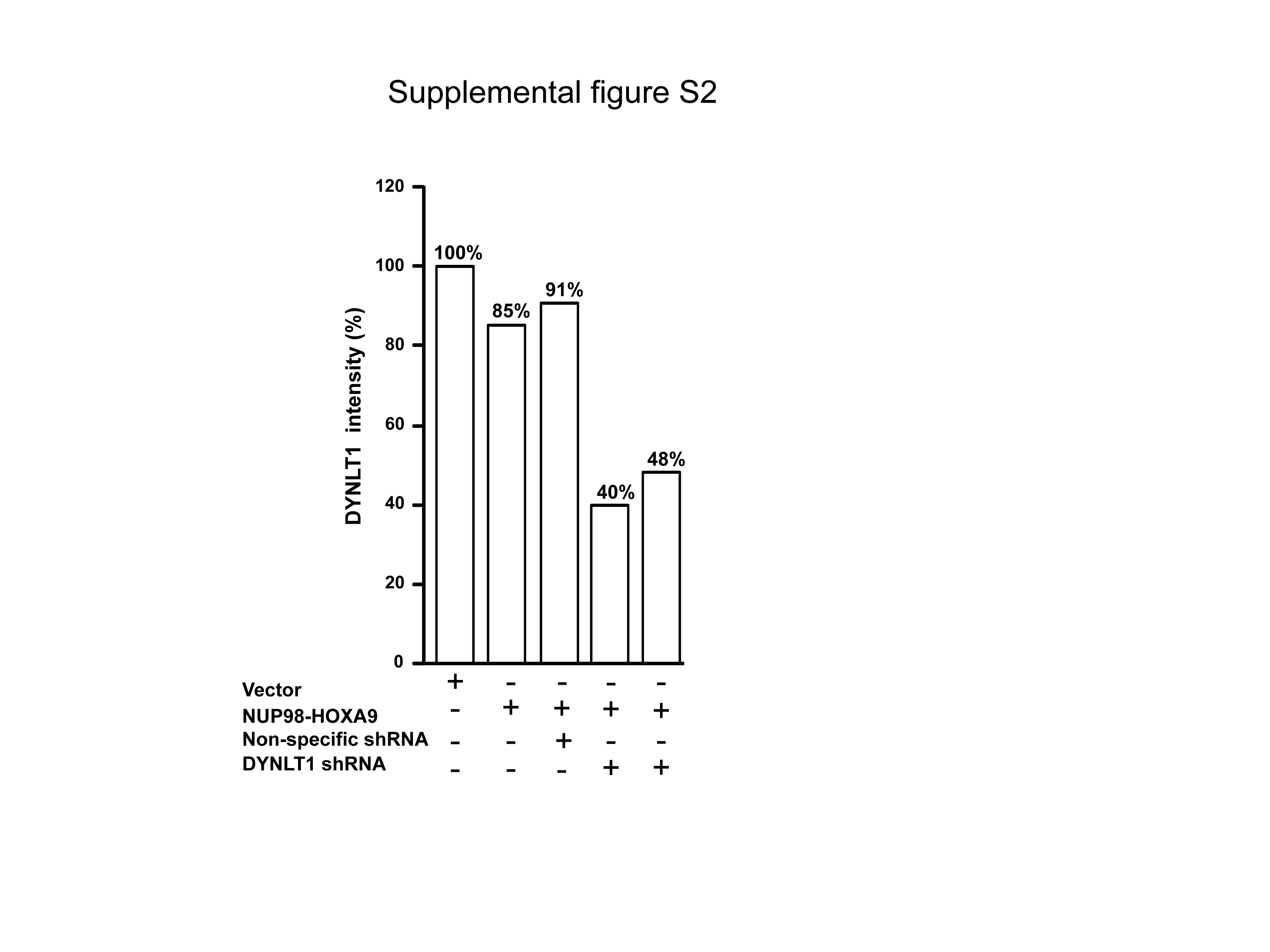

Supplement: Figure S2 — Densitometric quantification of shRNA-mediated DYNLT1 knockdown. K562 cells were transfected with a firefly luciferase construct driven by the KBTBD10 promoter with either empty vector or vector expressing NUP98-HOXA9. In addition, the transfections included either empty vector, or vector expressing non-specific shRNA, or vectors expressing 2 different DYNLT1 shRNAs. The intensities of the DYNLT1 bands in Fig. 7 were quantified using Chemidoc Quantity One software (BioRad). The DYNLT1 band intensities are shown as % of the empty vector control in lane 1. (TIF) [file pone.0067032.s002.tif]
